# Evaluating BG-Sentinel trap setting as an effective surveillance tool for mosquito vectors in the Republic of Cyprus

**DOI:** 10.1051/parasite/2026033

**Published:** 2026-06-03

**Authors:** Georgios Balatsos, Vasileios Karras, Marco Neira, Renaud Lancelot, Christiana Antoniou, Avgoustinos S. Stephanou, Yiannis Pigkeridis, Kyriakos Papazacharia, Giorgos Papazacharia, Marios Violaris, Maria Sakellariou Sofianou, Marina Bisia, Jeremy Bouyer, Antonios Michaelakis

**Affiliations:** 1 Laboratory of Insects & Parasites of Medical Importance, Benaki Phytopathological Institute Kifisia Greece; 2 Climate and Atmosphere Research Center (CARE-C), The Cyprus Institute Nicosia Republic of Cyprus; 3 UMR Astre, Cirad, Inrae, Univ. Montpellier, Plateforme Technologique Cyroi Ste Clotilde La Réunion France; 4 Medical Entomology Laboratory, Ministry of Health Republic of Cyprus

**Keywords:** Dry ice, BG-lure, *Aedes albopictus*, *Aedes aegypti*, Larnaca, Nicosia

## Abstract

Effective surveillance of invasive *Aedes* mosquitoes, particularly *Ae. aegypti* and *Ae. albopictus*, is essential for early detection, risk assessment, and optimization of control strategies such as the Sterile Insect Technique (SIT). Although BG-Sentinel 2 traps are widely used in Europe, their performance under eastern Mediterranean conditions remains insufficiently evaluated. We assessed the attractiveness of BG-Sentinel 2 traps baited with BG-Lure, dry ice (CO_2_), their combination, and unbaited controls using a Latin square field design in Larnaca and Nicosia, Cyprus. A total of 1,649 mosquitoes were collected. In Larnaca, *Ae. aegypti* occurred at low densities. Male captures were consistently low and unaffected by attractants, while female captures increased significantly only when BG-Lure and CO_2_ were combined (RR = 2.7, 95% CI: 1.6–4.5). In Nicosia, *Ae. albopictus* was abundant, but responses were sex-specific. Male captures were significantly reduced by CO_2_ alone (RR = 0.297, 95% CI: 0.175–0.467), with a weaker reduction when combined with BG-Lure, while female captures were not significantly affected by any treatment. For *Culex pipiens*, CO_2_ strongly increased female captures (RR ≈ 16), irrespective of BG-Lure, whereas males showed only a modest response to BG-Lure alone. Overall, attractant efficacy was highly species- and sex-specific. Standard baiting strategies did not consistently enhance detection of invasive *Aedes* mosquitoes and, in some cases, reduced male captures, with implications for SIT programs. These findings emphasize the need for local validation of surveillance protocols particularly at points of entry.

## Abstract (fr)

Une surveillance efficace des moustiques invasifs du genre *Aedes*, en particulier *Ae. aegypti* et *Ae. albopictus*, est essentielle pour la détection précoce, l’évaluation des risques et l’optimisation des stratégies de lutte telles que la technique de l’insecte stérile (TIS). Bien que les pièges BG-Sentinel 2 soient largement utilisés en Europe, leur performance dans les conditions de la Méditerranée orientale demeure insuffisamment évaluée. Nous avons étudié l’attractivité des pièges BG-Sentinel 2 appâtés avec du BG-Lure, du dioxyde de carbone (CO_2_, sous forme de glace carbonique), leur combinaison, ainsi que des témoins non appâtés, à l’aide d’un dispositif expérimental en carré latin à Larnaca et Nicosie (Chypre). Un total de 1 649 moustiques a été collecté. À Larnaca, *Ae. aegypti* était présent à faible densité. Les captures de mâles sont restées faibles et n’ont pas été influencées par les attractifs, tandis que les captures de femelles n’ont augmenté significativement que lorsque le BG-Lure et le CO_2_ étaient combinés (RR = 2,7 ; IC à 95 % : 1,6–4,5). À Nicosie, *Ae. albopictus* était abondant, mais les réponses aux attractifs étaient spécifiques au sexe. Les captures de mâles ont été significativement réduites par le CO_2_ seul (RR = 0,297 ; IC à 95 % : 0,175–0,467), avec un effet négatif atténué en présence du BG-Lure, tandis que les captures de femelles n’ont pas été significativement affectées par les traitements. Pour *Culex pipiens*, le CO_2_ a fortement augmenté les captures de femelles (RR ≈ 16), indépendamment du BG-Lure, alors que les mâles n’ont montré qu’une réponse modeste au BG-Lure seul. Globalement, l’efficacité des attractifs dépendait fortement de l’espèce et du sexe. Les stratégies d’appâtage standard n’ont pas systématiquement amélioré la détection des *Aedes* invasifs et ont parfois réduit les captures de mâles, un élément critique pour les programmes fondés sur la TIS. Ces résultats soulignent la nécessité de valider localement les protocoles de surveillance, en particulier aux points d’entrée.

## Introduction

The global expansion and establishment of *Aedes* invasive mosquito (AIM) species in new geographic areas present a growing threat to public health worldwide due to the role of this species in the transmission of arboviruses such as dengue, chikungunya, Zika, and yellow fever. In recent decades, Europe has increasingly faced challenges with AIM species, introduced primarily through global trade and travel [32, 53]. While several AIM species are sporadically imported, only four have successfully established across Europe: the Japanese bush mosquito *Aedes (Ae.) japonicus*, Theobald, 1901), the Korean bush mosquito (*Aedes (Ae.) koreicus*, Edwards, 1917), the Asian tiger mosquito (*Aedes (Ae.) albopictus*, Skuse, 1894), and dengue mosquito or yellow fever mosquito (*Aedes (Ae.) aegypti*, Linnaeus, 1762) [15, 24, 32, 53]. These *Aedes* species exhibit high invasive potential, primarily driven by their ecological adaptability, especially their capacity to produce drought-resistant diapausing eggs, while *Ae. aegypti* can additionally persist through quiescence [17, 18, 32, 58]. The pathways of introduction and spread are scale-dependent and closely linked to human activity. At the global level, passive transport via maritime freight, especially in association with goods such as used tires and ornamental plants (e.g., lucky bamboo), is considered the primary route of introduction [28, 33]. Aircraft also contribute significantly to long-distance dispersal. Within Europe, national and regional spread is largely driven by ground transportation, particularly through vehicles and trains, as these species are frequently transported inadvertently with human movement [24, 28, 32, 38, 59].

In addition, the establishment and spread of AIM further highlights the urgent need for strong surveillance systems at high-risk points of entry and rapid emergency response. *Aedes japonicus* has significantly expanded its range and now is established in many European countries, including Austria, Belgium, France, Germany, the Netherlands, Switzerland, Slovenia, and Spain [1, 10, 15, 26, 27, 36]. Similarly, *Ae. koreicus*, although still restricted, has been reported in several European countries including Belgium, Italy, Slovenia, Hungary, and Germany [7, 39, 48].

Among the invasive mosquito species introduced to Europe*, Ae. albopictus* and *Ae. aegypti* are among the most concerning due to their proven roles as vectors of dengue, yellow fever, chikungunya, and Zika arboviruses [29]. *Aedes albopictus* has achieved extensive global distribution, including in North and South America and Europe, representing the widest distribution ever recorded for the species [21, 53]. Since July 2024, *Ae. albopictus* has newly become classified as established in the Republic of Cyprus and Slovakia [54]. Despite its long-standing presence in parts of southern Europe, the species distribution remains dynamic, with continued expansion across the continent. Additionally, sporadic introductions without confirmed establishment have been reported in Czechia, Liechtenstein, the Netherlands and Sweden, highlighting the current risk of further geographical expansion facilitated by human-mediated transport and increasing climatic suitability [21]. *Aedes aegypti* was widely distributed across southern Europe, the Near East, and North Africa from the late 18th century until the mid-20th century [22, 61]. During this period, the mosquito was responsible for significant disease burden, including a severe dengue outbreak in Athens in 1927–1928 [45]. However, the species largely disappeared from continental Europe by the 1950s. Importantly, *Ae. aegypti* has since re-established populations in the Republic of Cyprus and self-sustaining populations in several outermost EU regions, notably Madeira (Portugal) and La Reunion, France as well as in the neighboring countries Türkiye and Egypt [22, 28, 49, 54]. The Republic of Cyprus, with its warm Mediterranean climate and strategic geographic position, has long been identified as a high-risk area for AIM introduction. Despite early surveillance efforts initiated in 2019, *Ae. albopictus* was not detected until October 2022 in the port area of Limassol, where it was subsequently confirmed in nearby municipalities of Mesa Geitonia and Germasogeia. In parallel, *Ae. aegypti* was recorded in November 2021 in the Larnaca district, marking its first appearance in the Republic of Cyprus since 1959 and its confirmed overwintering in 2022 [40, 54]. Currently in Cyprus, *Ae. albopictus* is established in multiple urban areas, whereas *Ae. aegypti* remains spatially restricted to Larnaca District under active control.

Whereas *Ae. albopictus* has been the vector in recent European mosquito-borne disease outbreaks of Dengue and chikungunya, the reestablishment of *Ae. aegypti* poses a higher risk for public health mainly due to the fact that it is consider the primary vector for these diseases [47, 50]. Thus, the recent reintroduction of *Ae. aegypti* and *Ae. albopictus* in the Republic of Cyprus warrants the establishment of an efficient surveillance system for both species, to support the surveillance program and vector control interventions. Following the detection of both species and with the technical guidance of the International Atomic Energy Agency (IAEA), the Republic of Cyprus initiated a coordinated emergency response against *Ae. aegypti* [12, 54]. As a result, in April 2023, a contingency plan was prepared by the IAEA and adopted by the Ministry of Health, aiming to eradicate *Ae. aegypti* from Larnaca District. Among others, the contingency plan recommended the establishment of a national surveillance system, an enhanced vector control program with biocides, a door-to-door campaign, and an environment-friendly control program employing the Sterile Insect Technique (SIT) [12, 54] [4, 5]. The pilot SIT trial was implemented in Kiti, Larnaca district, with promising initial outcomes. Its continuation requires accurate baseline data on the wild population density, and in parallel the assessment of the survival and dispersal of sterile males [3].

Evaluating the performance of commonly used adult traps, such as the BG-Sentinel 2 trap (Biogents, Regensburg, Germany) baited with dry ice and/or BG-Lure, to sample *Ae. aegypti* and *Ae. albopictus* under different environmental settings, is essential for enhancing vector surveillance and control strategies. While several studies have evaluated the performance of the BG-Sentinel 2 trap, such as in La Reunion, no studies have been conducted in a Mediterranean country where *Ae. aegypti* and *Ae. albopictus* occur under the same climate and environmental conditions [14, 42, 44]. The results are intended to recommend the most effective monitoring strategy to be used in future SIT interventions in the Republic of Cyprus, where an effective male trapping system is a critical component to estimate the sterile-to-wild male ratio, sterile male survival and distribution in the target area, and program success. Additionally, the results may guide the selection of optimal trap configurations and attractants for entomological surveillance programs in other European countries, particularly at points of entry (PoEs), to enhance the early detection and monitoring of invasive mosquito species and allow a rapid emergency response [35]. This is of paramount importance for European countries implementing active surveillance programs at PoEs that have connections with countries where *Ae. aegypti* is established. Enhancing the early detection of invasive mosquito species by adopting efficient surveillance systems and tools is crucial to implement rapid interventions measures, thereby mitigating the risk of establishment and spread within Europe. Therefore, the present study aimed to validate the efficacy of BG-Sentinel 2 traps using various baiting combinations; BG-Lure (human skin scent mimics), dry ice (CO_2_) and their combination compared to unbaited traps, for the surveillance of adult *Ae. aegypti, Ae. albopictus*, and native *Culex (Cx.) pipiens* using a Latin square field design.

## Methods

### Study areas

**Figure 1:**
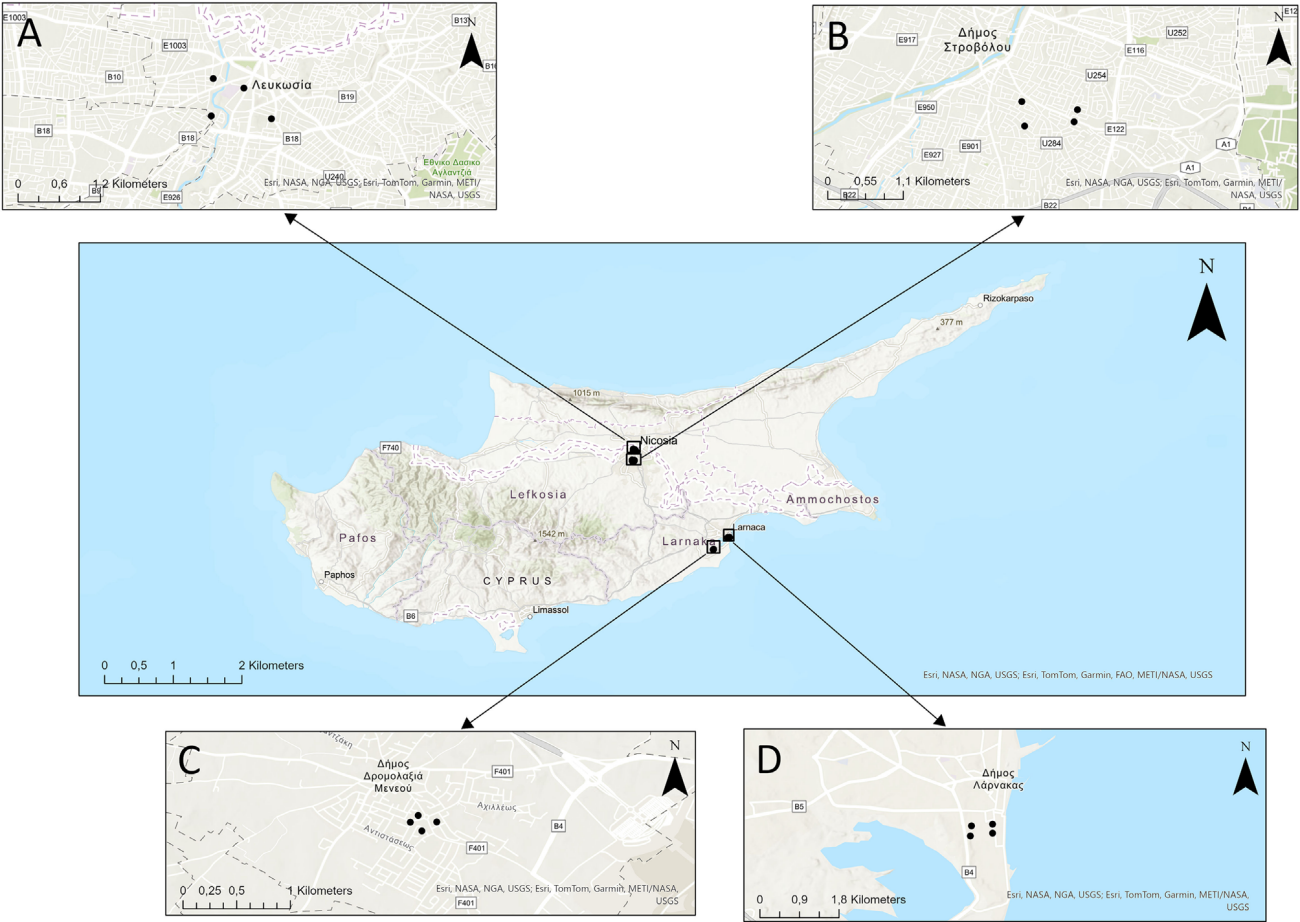
Map of the Republic of Cyprus showing the study regions and sampling sites. Each region (Nicosia and Larnaca) included two sampling sites, where four trap configurations were deployed at each site. Panels A and B show the locations and distribution of sampling points in Nicosia, specifically in the municipalities of Nicosia and Strovolos. Panels C and D show the locations and distribution of sampling points in Larnaca, specifically in the municipalities of Dromolaxia–Meneou and Larnaca.

Field trials were conducted in the Republic of Cyprus from October 20 to November 14, 2024, in the cities of Nicosia and Larnaca (Fig. 1). This study period was selected because it aligns with the high-density period for *Ae. aegypti* in Larnaca and captures the secondary late-autumn surge of *Ae. albopictus* observed in Nicosia, following its primary peak in late summer (unpublished data). Nicosia (35.1856° N, 33.3823° E) is the capital of the Republic of Cyprus, situated at approximately 160 m above sea level. This city experiences a semi-arid Mediterranean climate with hot, dry summers, and mild winters [23]. Sampling was conducted in urban residential areas characterized by irrigated gardens and domestic water containers, where *Ae. albopictus* has recently established.

Larnaca (34.918394° N, 33.620065° E) is a coastal city located at approximately 0 to 26 m above the sea level, with a hot-summer Mediterranean climate, high relative humidity during the warm season, and mild winters [23]. Sampling sites in Larnaca were in residential peripheral (semi-urban/suburban) areas characterized by a low density of inhabitants and numerous potential breeding sites, supporting large populations of container-breeding *Aedes* mosquitoes. Specifically, the Dromolaxia–Meneou and Mackenzie areas are characterized by predominantly detached housing, limited high-rise development, and abundant outdoor spaces such as yards, gardens, and auxiliary structures that favor the presence of artificial containers.

In each location, two study sites with similar environmental conditions were selected, each comprising four BG-Sentinel 2 trap sampling stations, yielding a total of eight trap sampling points. The selected sites shared comparable urban land use, vegetation cover, and climatic conditions. Sampling sites in both Larnaca and Nicosia were selected based on prior surveillance data to maximize the detection density, with locations in Larnaca emphasizing proximity to previously identified *Ae. aegypti* hotspots and those in Nicosia emphasizing areas near known *Ae. albopictus* hotspots. Due to the absence of *Ae. albopictus* in Larnaca and *Ae. aegypti* in Nicosia, mosquito samples were transported to field laboratories in the city where they were collected and processed there, to avoid the risk of introducing either species to areas where they are not currently present.

### Study design

The study areas and period (autumn months) were selected to agree with the peak activity of both *Ae. aegypti* and *Ae. albopictus*, based to the available routine surveillance data from the Ministry of Health. Consequently, our samplings ensure that the collected data are representative of the highest population densities and maximize the sensitivity of the study for both male and female mosquitoes.

Four BG-Sentinel 2 trap configurations were evaluated using a Latin square design, as described in Table 1. To avoid cross-contamination of any residual attractants between experimental conditions, all traps were brand new at the beginning of the study, and each trap was only used for a specific type of attractant configuration throughout the study.

**Table 1:** Descriptions of BG-Sentinel trap configurations tested in the field experiment.

Trap Label	Trap configuration	Attractants
D	Trap baited with dry ice	CO_2_
DL	Trap baited with dry ice and BG Lure	CO_2_ + BG Lure*
L	Trap baited with only BG Lure	BG Lure*
N	Negative control (trap without any bait)	None

*BG Lure cartridge: Commercially available synthetic mosquito attractant (Biogents).

At each sampling site, the four trap configurations were deployed simultaneously in a clockwise arrangement, ensuring a minimum inter-trap distance of 90 m to minimize bait interference [19]. Traps were placed in the peri-domiciliary areas of local residences, in shaded, rain-protected, and secure locations with prior consent from residents. To standardize micro-environmental conditions as much as possible, traps were typically positioned near vegetation or other sheltered micro-habitats, avoiding direct sunlight, strong wind exposure, and obvious breeding containers whenever possible. Traps using CO_2_ as attractant were loaded with 4 kg of dry ice pellets contained in an insulated cooler bag placed inside the trap. This quantity of dry ice was sufficient to supply CO_2_ for 24 h.

Each baiting treatment was rotated among the four trap positions every 24 h according to a Latin square design. This ensured that each trap position received each baiting treatment once during a complete 4-day sampling block. The 4-day rotation was repeated for four consecutive weeks, resulting in a total of 16 observations per trap configuration and position. This design controlled for environmental variation associated with trap location and sampling date, which served as proxies for local habitat conditions and short-term weather factors such as temperature, humidity, and wind [14].

At the end of each 24-hour sampling period, collection nets from the BG-Sentinel 2 traps were retrieved, transported to the respective laboratories, and stored at −20 °C to kill the mosquito specimens. Mosquitoes captured in the traps during the study period were identified to species level using taxonomic keys [9, 16], counted and recorded by sex in a relational database management system for subsequent analysis.

### Data analysis

In this study, we addressed the primary question of which combination of dry ice and BG-Lure maximizes BG-Sentinel 2 trap performance (*i.e.*, the optimized baiting configuration). We fitted a separate Bayesian mixed-effect Poisson model for each combination of species (*Ae. albopictus, Ae. aegypti* or *Cx. pipiens*) and sex (male or female), the latter because males and females are not attracted to the traps for the same cues: seeking hosts for females, mating for males. Each model had the same fixed effects: dry ice (coded yes/no), BG Lure (coded yes/no), and their interaction. Two random effects – assumed to be independently and identically distributed, were associated with the model intercept, to account for random fluctuations in mosquito density by replicate and location. Posterior distributions of the fitted values were used to estimate relative risks (RR) compared with the unbaited control configuration. Posterior distributions from the Bayesian mixed-effects models were used to estimate the expected number of mosquitoes captured per trapping session for each baiting configuration. In this study, we refer to this model-based expected count as the density of mosquitoes, expressed as the predicted mean number of individuals collected per BG-Sentinel 2 trap per 24-hour trapping session. We use the term density in a relative ecological sense, as an index of local mosquito abundance rather than an absolute estimate of population size per unit area (Table S1). These RR quantified the change in expected mosquito captures when BG-Sentinel 2 traps were baited with dry ice alone, BG-Lure alone, or both attractants combined. Data analysis, was performed within the R software environment for statistical computing and graphics [43]. Bayesian models were fitted using the integrated Laplace approximation (inla) of the Markov chain process [46]. Throughout this study, we set *α* = 0.05. For completeness, fitted model coefficients are shown in the Supplementary Material.

Furthermore, ecological community parameters were calculated using standard biodiversity indices. Species richness was estimated using the Margalef index, diversity using the Shannon index (*H*′), and dominance using the Simpson index, all computed with the vegan package in R (Oksanen *et al.*, 2013) [37]. Pielou’s evenness index was calculated as *E_h_* = *H*′/ln*S*, where *H*′ is the Shannon diversity index and *S* is the number of species [41].

In addition, weather data during the sampling period were obtained from the Department of Meteorology of the Republic of Cyprus. Data were collected from the Automatic Weather Stations (AWS) located in Athalassa (Nicosia) (35° 08′ 27.50″ N, 33° 23′ 47.85″ E), and at the Larnaca Airport (34° 52′ 24.71″ N, 33° 37′ 02.57″ E). Both stations are located approximately 3.5 km from the corresponding city’s sampling blocks. We collected data on environmental variables known to influence mosquito activity (and consequently trap catch variability), including temperature, humidity, and wind speed.

## Results

A total of 1,649 mosquitoes were collected during the field trials. In Nicosia, the mosquito populations were dominated by *Cx. pipiens* (*n* = 702, females: 58.0% / males: 3.8%) and *Ae. albopictus* (*n* = 421, females: 23.4% / males: 13.6%). In Larnaca, *Cx. pipiens* (*n* = 349, females: 48.8% / males: 20.2%) and *Ae. aegypti* (*n* = 133, females: 21.9% / males: 4.3%) were the predominant species. Other species identified included *Cx. theileri, Cx. perexiguus*, and *Culiseta longiareolata* in Nicosia, *while Cx. theileri*, *Cx. perexiguus*, and *Ae. caspius* were present in Larnaca (Table 2 and Table S2). Notably, *Ae. cretinus* was not detected in either study area during this period.

**Table 2:** Total number of mosquitoes captured per species in each study area (Nicosia and Larnaca) from October 20 to November 14, 2024 (*Cx. = Culex*, *Ae. = Aedes*, *Cs. = Culiseta*).

	Mosquito species/sex (total number)											Shannon Index (*H*′)	Evenness (*E*)
*Study area*	*Cx. pipiens females*	*Cx. pipiens* males	*Cx. theileri* females	*Cx. theileri* males	*Cx. perexiguus* females	*Ae. aegypti* females	*Ae. aegypti* males	*Ae. caspius* females	*Ae. albopictus* females	*Ae. albopictus* males	*Cs. longiareolata* females		
Nicosia	659	43	5	0	4	0	0	0	266	155	4	0.729	0.453
Larnaca	247	102	9	5	5	111	22	5	0	0	0	0.798	0.496

The species composition differed between the two study areas. In Nicosia, the community was strongly dominated by *Cx. pipiens* and *Ae. albopictus*, resulting in a Shannon diversity index of *H*′ = 0.729. Although Nicosia had a higher total mosquito abundance, its lower evenness (*E* = 0.453) reflects the marked dominance of these two species. In contrast, Larnaca exhibited slightly higher diversity (*H*′ = 0.798) and evenness (*E* = 0.496), supported by a more balanced distribution among *Cx. pipiens*, *Ae. aegypti*, and several secondary species (Table 2). Subsequently, we focused on evaluating the attractiveness of baited BG-Sentinel 2 traps to (i) *Ae. aegypti* in Larnaca, (ii) *Ae. albopictus* in Nicosia, and (iii) *Cx. pipiens* in both regions.

### *Aedes aegypti* (Larnaca)

**Figure 2:**
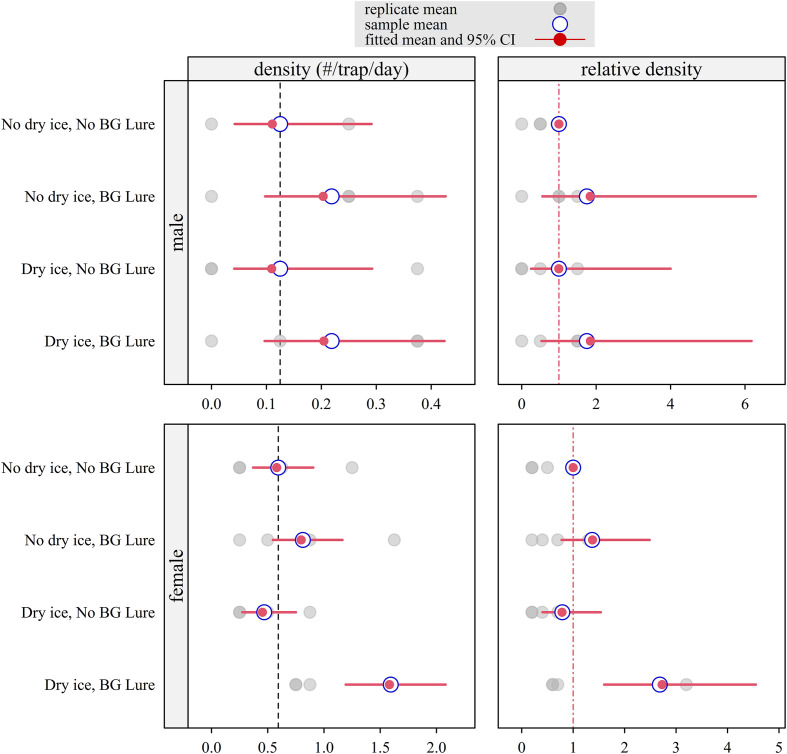
Model-estimated density and relative density of *Aedes aegypti* fitted with a Bayesian Poisson model with data collected during a field trial with a Latin square design in Larnaca (Cyprus), October–November 2024 (*n* = 8 trapping sessions for each grey dot). Separate models were fitted for males and females. Density represents the posterior mean expected number of mosquitoes captured per BG-Sentinel 2 trap per 24-hour trapping session. Relative density represents the ratio of this expected count to that of the control group (no dry ice, no BG-Lure), which was set to 1. Red segments indicate 95% CI. For relative density, intervals crossing the vertical dashed red line at 1 indicate that the null hypothesis of no difference from the control cannot be rejected (*α* = 0.05).

The surveillance efficacy for *Ae. aegypti* is summarized in Figure 2. For males, the density remained consistently low across all trap configurations, with a mean capture rate ranging from 0.13 (95% CI: 0.04–0.3; dry ice only) to 0.2 (95% CI: 0.1–0.4; dry ice + lure). No significant differences were observed between trap configurations for males.

In contrast, female densities were significantly higher. A synergistic effect was observed when combining dry ice and BG-Lure, resulting in a significant increase in capture efficacy with an RR of 2.7 (95% CI: 1.6–4.5) compared to the unbaited control.

### *Aedes albopictus* (Nicosia)

**Figure 3:**
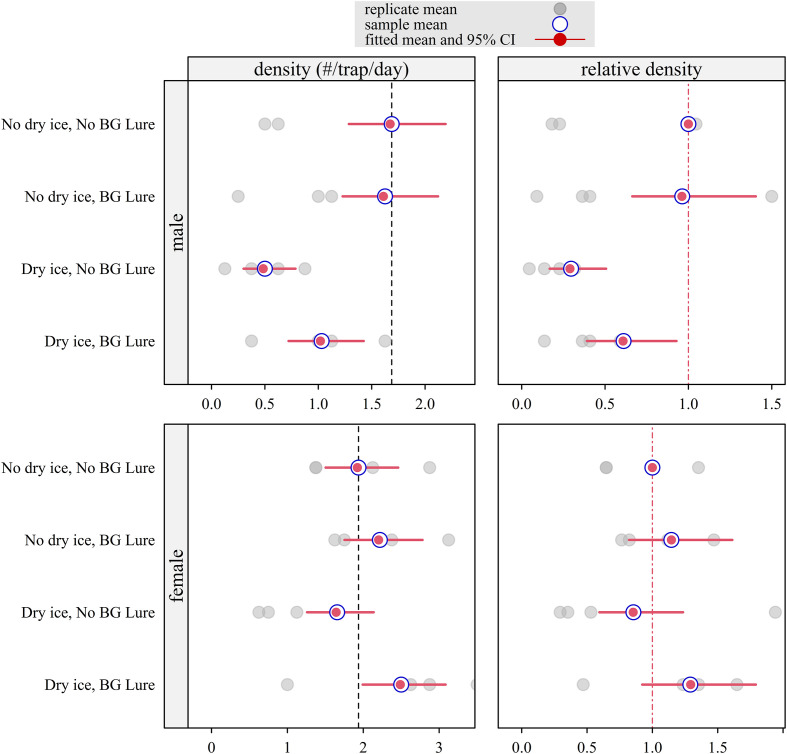
Model-estimated density and relative density of *Aedes albopictus* fitted with a Bayesian mixed-effect Poisson model (males), or a Bayesian marginal negative binomial model (females) of attractiveness implemented in a field trial with a Latin square design in Nicosia (Cyprus), October–November 2024 (*n* = 8 trapping sessions for each combination of sex, dry ice, and lure). Separate models were fitted for males and females. Density represents the posterior mean expected number of mosquitoes captured per BG-Sentinel 2 trap per 24-hour trapping session. Relative density represents the ratio of this expected count to that of the control group (no dry ice, no BG-Lure), which was set to 1. Red segments indicate 95% CI. For relative density, intervals crossing the vertical dashed red line at 1 indicate that the null hypothesis of no difference from the control cannot be rejected (*α* = 0.05).

Due to the high capture rates of *Ae. albopictus*, separate Bayesian models were utilized (Mixed-effect Poisson for males; Marginal Negative Binomial for females) (Fig. 3). For *Ae. albopictus*, sex-specific differences in response to attractants were observed. In males, the use of dry ice alone had a significant negative effect on capture efficacy (RR = 0.297, 95% CI: 0.175–0.467). This negative impact persisted, though less severely, when dry ice was combined with BG-Lure (RR = 0.612, 95% CI: 0.427–0.844). In contrast, no significant differences were detected between any of the attractant combinations and the control group for female *Ae. albopictus* in this trial.

### *Culex pipiens* (Nicosia and Larnaca)

**Figure 4:**
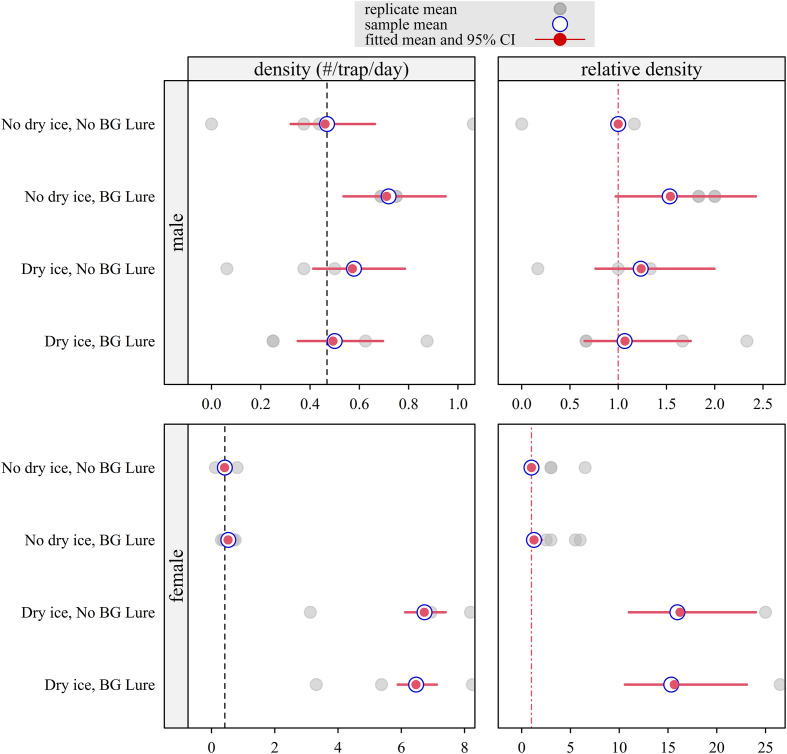
Density and relative density of *Cx. pipiens* fitted with a Bayesian mixed-effect Poisson model of BG-Sentinel 2 trap attractiveness on data collected during a field trial with a Latin square design in Larnaca and Nicosia (Cyprus), October–November 2024 (*n* = 8 trapping sessions for each combination of sex, dry ice, and lure). Separate models were fitted for males and females. Density represents the posterior mean expected number of mosquitoes captured per BG-Sentinel 2 trap per 24-hour trapping session. Relative density represents the ratio of this expected count to that of the control group (no dry ice, no BG-Lure), which was set to 1. Red segments indicate 95% CI. For relative density, intervals crossing the vertical dashed red line at 1 indicate that the null hypothesis of no difference from the control cannot be rejected (*α* = 0.05).

The response of *Cx. pipiens* showed distinct sexual dimorphism regarding attractant preference (Fig. 4). In males, the effects of dry ice were negligible; however, BG-Lure used alone resulted in a small but statistically significant increase in capture efficacy (RR = 1.533, 95% CI: 1.131–2.031). This positive effect was not observed when BG-Lure was combined with dry ice, suggesting that the presence of carbon dioxide neutralized the lure’s contribution. In contrast, females showed a strong and statistically significant positive response to dry ice, regardless of whether BG-Lure was present. Relative risks reached 15.95 (95% CI: 14.50–17.54) for dry ice alone and 15.32 (95% CI: 13.91–16.86) for the combined treatment. The addition of BG-Lure did not significantly improve performance beyond that achieved with dry ice alone.

### Environmental conditions

**Figure 5:**
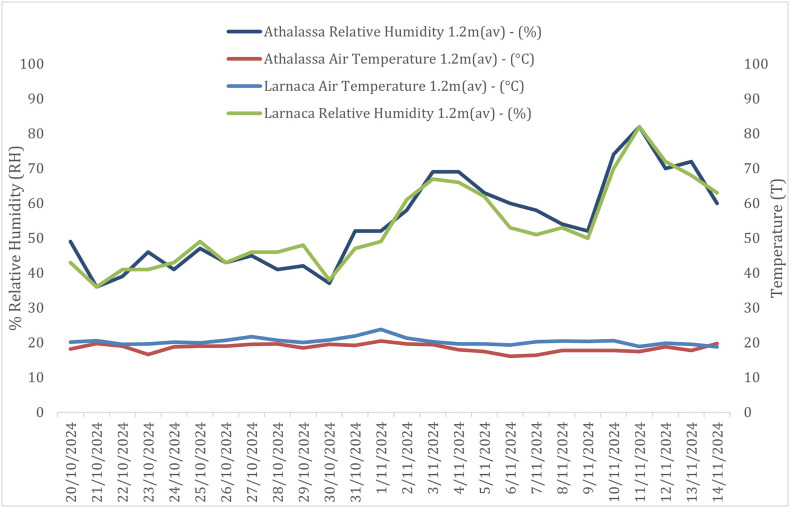
Environmental conditions in Nicosia and Larnaca during the sampling period. The graph shows the daily average temperature (°C) and relative humidity (%) recorded at the Athalassa and Larnaca airport, Automatic Weather Station. In Nicosia, the temperatures remained relatively stable between 20 and 24 °C, while humidity ranged from 40% to 80%. In Larnaca, the temperatures remained stable at approximately 20 °C, while humidity ranged from 40% to 80%.

**Figure 6:**
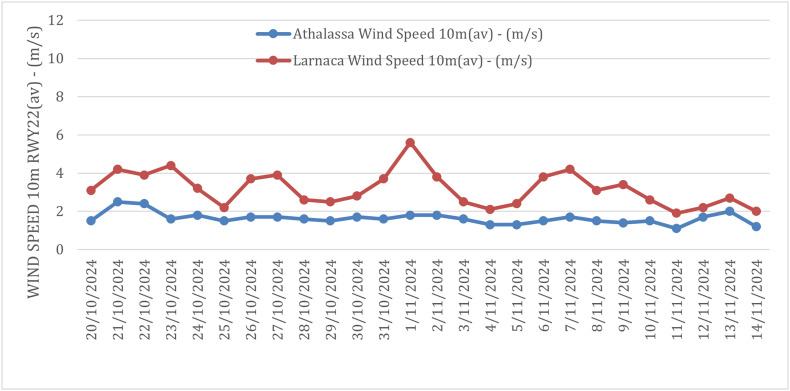
Wind conditions in Nicosia and Larnaca during the sampling period. The graph shows the recorded wind speeds, ranging from 1 Bft (average 0.9 m/s) to almost 2 Bft (average 2.9 m/s), measured at the Athalassa Automatic Weather Station. In Larnaca, the recorded wind speeds, which exhibited greater variation than in Nicosia, reaching up to 4 Bft (average 5.6 m/s), were measured at the Larnaca Airport Automatic Weather Station.

Throughout the sampling period, average temperatures in Nicosia remained stable between 20 and 24 °C, with humidity ranging from 40% to 80%, and wind speeds ranging from 1 Bft (average 0.9 m/s) to almost 2 Bft (average 2.9 m/s) (Figs. 1 and 2). In Larnaca, temperatures were similarly stable at approximately 20 °C, humidity ranged from 40% to 80%, and wind speeds showed greater variation, reaching up to 4 Bft (average 5.6 m/s) (Figs. 5 and 6).

## Discussion

The current study evaluated the performance of different baits for BG-Sentinel 2 traps in detecting medically important mosquito species under field conditions representative of southeastern Mediterranean urban environments. Our findings provide critical data for designing effective surveillance strategies and vector control programs including SIT, especially in newly colonized regions with limited historical data.

For *Ae. aegypti*, male capture rates were consistently low across all baiting treatments and no attractant combination produced statistically supported improvements relative to the unbaited control. Female capture rates were higher and the combination of dry ice and BG-Lure was associated with a statistically supported increase in trap catches compared with the control. These results suggest that, under the study conditions, combined host-associated cues can enhance female *Ae. aegypti* captures, whereas male detection remains challenging. From a surveillance perspective, low male capture rates imply that absence of captures cannot be interpreted as absence of the species when trap density is limited. Consequently, higher trap densities or complementary surveillance tools may be required, particularly during early invasion or post-suppression phases. The addition of dry ice as bait did not increase trap attractiveness for male *Ae. aegypti* in our study, a result that contrasts with previous research showing enhanced BG-Sentinel 2 trap performance when carbon dioxide is used as an attractant [8, 14, 60]. This discrepancy may reflect differences in local mosquito density, trap location, environmental conditions, and host availability, all of which can influence the strength and dispersion of attractants and thus mosquito responses in the field. It may also indicate that the uncontrolled and temporary flow of CO_2_ is not as attractive as continuous controlled release, which is possible with compressed CO_2_ [14].

Although dry ice alone did not significantly increase *Ae. aegypti* captures, its combination with BG-Lure did enhance female trap performance. This finding contrasts with other studies which report increased effectiveness of BG-Sentinel traps when baited with carbon dioxide [30, 42]. Considering that both *Ae. aegypti* and *Ae. albopictus* were absent from the Republic of Cyprus until very recently (and therefore the populations currently established on the island did not have much time to build up), the lack of benefit attributable to CO_2_ observed in our study might be partially explained by a low population density of these species, especially for *Ae. albopictus* which has already been established for many years in other Mediterranean countries. In the case of *Ae. aegypti*, the observed population levels are relatively low considering that the Cyprus Ministry of Health has implemented an immediate response, following its first detection, including door-to-door actions and SIT applications [13]. In addition, trap location may have influenced attractant performance, as mosquito host-seeking and resting behavior in these newly established populations is still being characterized under local environmental conditions.

Another factor that could affect the overall efficiency of the baits in Larnaca is the relatively windy conditions prevailing in this area, which during the sampling period reached up to 5.6 m/s, and never dipped below 2 m/s (Fig. 6). Wind speeds at this level can affect the linearity of the plume of attractant and reduce its efficiency. Importantly, previous studies have reported that wind speeds as low as 0.5 m/s can reduce mosquito trap catches by up to 50% [11]. Therefore, our results suggest that attractant effectiveness may vary across environmental contexts and highlight the importance of localized trap validation before implementation. Detailed information on habitat features, such as vegetation density, proximity to alternative hosts or breeding sites, and small-scale structural variation was not systematically recorded. These factors can influence BG-Sentinel 2 trap performance for *Ae. aegypti* and *Ae. albopictus* [51], and their inclusion in future studies could help further refine the interpretation and applicability of our findings.

Unlike *Ae. aegypti*, the detection of male *Ae. albopictus* was better explained by the full Poisson model, which revealed significant effects of the attractant type. Dry ice alone had a strong and statistically significant negative effect on trap attractiveness, whereas BG-Lure had a weaker, non-significant negative effect. These results imply a complex behavioral response of male *Ae. albopictus* to synthetic host cues, potentially influenced by competing environmental factors or local adaptation. These findings are particularly relevant for SIT programs, where accurate estimation of male abundance is essential for measuring sterile-to-wild ratios, which is fundamental to guide the field releases of sterile males. Therefore, BG Sentinel 2 traps are an appropriate tool for estimating sterile-to-wild male ratios, as a complementary approach to the human landing collection method [55]. A 2022 study from La Réunion reported that adding CO_2_ markedly increased male *Ae. albopictus* detection in BG-Sentinel traps, while BG-Lure provided no additional benefit [14]. These findings contrast with our results, where dry ice alone reduced male captures and the CO_2_–BG-Lure combination only partially improved performance. Several factors may explain these discrepancies, including differences in CO_2_ delivery systems (cylinders *vs.* dry ice), the environmental conditions between study sites, and potential strain-specific behavioral variation. Such differences highlight the need to better understand how attractant type and local ecological context influence male *Ae. albopictus* trap responsiveness, particularly for SIT applications. For female *Ae. albopictus*, neither attractant alone nor in combination significantly influenced trap catch, reinforcing the need for alternative or supplementary monitoring tools when targeting female *Ae. albopictus*. They also challenge the assumption that standard attractant-baited BG-Sentinel 2 traps are universally effective across mosquito taxa and sexes.

In parallel, *Cx. pipiens*, although native to Europe, remains a key target for surveillance due to its role in transmitting West Nile virus and other encephalitic arboviruses, and it is consistently the most abundant species detected in entomological surveillance. Its widespread presence, behavioral plasticity, and potential for overlapping habitats with *Aedes* species necessitate its inclusion in integrated vector surveillance programs [20, 52]. Moreover, the species occurs as two ecologically distinct biotypes, *pipiens* and *molestus* and their hybrids, which differ in host preference, diapause behavior, and reproductive strategies, thereby influencing pathogen transmission dynamics [25, 31]. In the surveillance of *Cx. pipiens* in Europe, the two forms are rarely distinguished and identified only using molecular techniques. The ornithophilic *pipiens* form sustains enzootic cycles of avian viruses, while the anthropophilic and autogenous *molestus* form, along with hybrids, may act as bridge vectors, facilitating spillover to humans and other mammals [2, 56, 57]. Its ability to exploit a broad range of larval habitats, including organically enriched urban environments, further supports its dominance in both rural and urban settings. Consequently, surveillance of *Cx. pipiens* not only provides essential insights into arbovirus dynamics, but also complements monitoring of invasive *Aedes* species within integrated vector management frameworks across Europe [20].

BG-Sentinel 2 traps are an operational tool for estimating sterile-to-wild male ratios in *Ae. albopictus* SIT programs, as shown in a Tirana mark-release-recapture (MRR) study where they achieved similar recapture performance to human landing catches (HLC) with 2.36% vs 1.57% [55]. However, the results may vary under different conditions, so they should be locally validated. It is also recommended that BG-Sentinel 2 trap ratios be complemented with HLC and egg hatch rates to better evaluate field competitiveness and program impact. Additionally, the outcomes from the current study demonstrate no significant effect of attractants on all species male (*Cx. pipiens* and *Aedes* spp.). Conversely, females showed a statistically significant positive response to dry ice, while BG-Lure had a weaker, non-significant effect for *Cx. pipiens*. These results suggest the use of CO_2_-baited traps for monitoring female *Cx. pipiens* populations, particularly in the context of West Nile virus risk assessment.

The biodiversity indices further highlight ecological differences between the two study areas that may influence mosquito surveillance outcomes [6]. Although overall mosquito abundance was higher in Nicosia, the lower Shannon diversity (*H*′ = 0.729) and evenness (*E* = 0.453) indicate a community strongly dominated by *Cx. pipiens* and *Ae. albopictus*. In contrast, Larnaca exhibited slightly higher diversity (*H*′ = 0.798) and evenness (*E* = 0.496), reflecting a more balanced distribution of species, but with the presence of more *Ae. aegypti* than *Ae. albopictus*. Areas with lower evenness are typically characterized by dominance of a few highly adapted species, which may intensify interspecific competition and influence host-seeking behaviour, and trap encounter rates. Conversely, more even communities may reflect a broader range of ecological niches and host-use patterns, potentially affecting how different species respond to synthetic attractants [34].

In conclusion, our results suggest that attractant efficacy is both species- and sex-specific and can be modulated by environmental factors. Therefore, reliance on standardized trap configurations may compromise the sensitivity of surveillance efforts. This is particularly relevant for *Ae. aegypti*, which is currently under re-establishment in the Republic of Cyprus after decades of absence. Under such low-population conditions, the probability of detection remains limited. This outcome should not be recorded only as a constraint, since it may simulate an invasion at an early stage at PoEs, offering valuable information for the available surveillance and control tools under introduction scenarios [3]. Additionally, this is particularly important for evaluating the suppression and potential eradication efforts in SIT programs currently targeting *Ae. albopictus*, and in the future, potential expansion of *Ae. aegypti* across Europe and the Mediterranean region.

## Supplementary material

*Supplemental File*: (Model coefficients) Mixed-effect “Poisson model of density of male *Aedes aegypti*” and Mixed-effect “Poisson model of density of female *Aedes aegypti*”. *Table S1:* Density and relative density of *Aedes aegypti* fitted with a Bayesian mixed-effect Poisson model, with data collected during a field trial with a Latin square design in Larnaca (Cyprus), October–November 2024. *Table S2:* Number of mosquitoes collected with each trap configuration in Larnaca and Nicosia, Cyprus. The supplementary material of this article is available at https://www.parasite-journal.org/10.1051/parasite/2026033/olm.
