# Supplementary material for: Evaluating BG-Sentinel trap setting as an effective surveillance tool for mosquito vectors in the Republic of Cyprus
Source: Parasite. 2026 Jun 3;33:31. doi: 10.1051/parasite/2026033 (PMC13233028; doi:10.1051/parasite/2026033)
Supplement: Supplementary file 1 — Supplemental File: (Model coefficients) Mixed-effect “Poisson model of density of male Aedes aegypti” and Mixed-effect “Poisson model of density of female Aedes aegypti”. [file parasite-33-31-s1.pdf]

Supplementary material

Model coefficients

Mixed-effect ¨Poisson model of density of male *Aedes aegypti*

```
Fixed effects:
                     mean    sd 0.025quant 0.5quant 0.975quant   mode kld
(Intercept)           -2.204 0.500     -3.185   -2.204     -1.224 -2.204   0
driceDry ice           0.000 0.707     -1.386    0.000      1.386  0.000   0
lureBG Lure            0.613 0.626     -0.616    0.613      1.841  0.613   0
driceDry ice:lureBG Lure  0.000 0.886     -1.737    0.000      1.737  0.000   0

Model hyperparameters:
                      mean       sd 0.025quant 0.5quant 0.975quant    mode
Precision for loc  22015.56 24148.38    1465.38 14443.49   86131.18 3993.99
Precision for repl 22020.64 24150.79    1466.57 14448.32   86142.80 3997.66

Deviance Information Criterion (DIC) ...............: 130.38
Deviance Information Criterion (DIC, saturated) ....: 89.00
Effective number of parameters .....................: 3.81

 is computed
```

Mixed-effect ¨Poisson model of density of female *Aedes aegypti*

```
Fixed effects:
                     mean    sd 0.025quant 0.5quant 0.975quant   mode kld
(Intercept)           -0.713 0.370     -1.464   -0.707      0.000 -0.707   0
driceDry ice          -0.243 0.345     -0.920   -0.243      0.434 -0.243   0
lureBG Lure            0.321 0.302     -0.271    0.321      0.913  0.321   0
driceDry ice:lureBG Lure  0.926 0.421      0.101    0.926      1.752  0.926   0

Model hyperparameters:
                   mean    sd 0.025quant 0.5quant 0.975quant mode
Precision for loc  10.89 10.37       1.88     7.88      38.39 4.43
```

Precision for repl 8.16 7.69 1.19 5.95 28.52 3.08

Deviance Information Criterion (DIC) ....: 315.52
Deviance Information Criterion (DIC, saturated) ....: 188.73
Effective number of parameters ....: -12.27

is computed
