# Supplementary material for: Evaluating BG-Sentinel trap setting as an effective surveillance tool for mosquito vectors in the Republic of Cyprus
Source: Parasite. 2026 Jun 3;33:31. doi: 10.1051/parasite/2026033 (PMC13233028; doi:10.1051/parasite/2026033)
Supplement: Supplementary file 2 — Table S1: Density and relative density of Aedes aegypti fitted with a Bayesian mixed-effect Poisson model, with data collected during a field trial with a Latin square design in Larnaca (Cyprus), October–November 2024. [file parasite-33-31-s2.pdf]

Table S1 Density and relative density of *Aedes aegypti* fitted with a Bayesian mixed-effect Poisson model, with data collected during a field trial with a Latin square design in Larnaca (Republic of Cyprus), October-November 2024 (n=8 trapping sessions for each combination of sex, dry ice, and lure). Separate models were fitted for males and females. For relative densities, the reference was the control group (no dry ice, no BG Lure).

					estimate and 95% credible interval (CI)			
type	sex	drice	lure	sessions	observed	fitted	lower	upper
density	female	No dry ice	No BG Lure	32	0.59	0.58	0.37	0.90
			BG Lure	32	0.81	0.80	0.55	1.16
		Dry ice	No BG Lure	32	0.47	0.45	0.27	0.75
			BG Lure	32	1.59	1.58	1.19	2.08
	male	No dry ice	No BG Lure	32	0.12	0.11	0.04	0.29
			BG Lure	32	0.22	0.20	0.10	0.43
		Dry ice	No BG Lure	32	0.12	0.11	0.04	0.29
			BG Lure	32	0.22	0.20	0.10	0.42
relative density	female	No dry ice	No BG Lure	32	1.00	1.00	1.00	1.00
			BG Lure	32	1.37	1.38	0.77	2.49
		Dry ice	No BG Lure	32	0.79	0.78	0.40	1.54
			BG Lure	32	2.68	2.73	1.60	4.55
	male	No dry ice	No BG Lure	32	1.00	1.00	1.00	1.00
			BG Lure	32	1.75	1.84	0.56	6.29

Dry ice	No BG Lure	32	1.00	1.00	0.25	4.00
	BG Lure	32	1.75	1.85	0.53	6.18
