# Supplementary material for: Evaluating BG-Sentinel trap setting as an effective surveillance tool for mosquito vectors in the Republic of Cyprus
Source: Parasite. 2026 Jun 3;33:31. doi: 10.1051/parasite/2026033 (PMC13233028; doi:10.1051/parasite/2026033)
Supplement: Supplementary file 3 — Table S2: Number of mosquitoes collected with each trap configuration in Larnaca and Nicosia, Cyprus. [file parasite-33-31-s3.pdf]

Table S2 Number of mosquitoes collected by each trap configuration in Larnaca and Nicosia, Cyprus.

[F, females; M, males; CO_2 = carbon dioxide supplied as dry ice; BG Lure = BG Lure cartridge: Commercially available synthetic mosquito attractant (Biogents AG, Germany); CO_2 + BG Lure = combined use of dry ice and BG Lure; None = negative control (trap without any bait)]

**Sampling area**	**Attractant**	***Culex pipiens* F**	***Culex pipiens* M**	***Culex theileri* F**	***Culex theileri* M**	***Culex perexiguus* F**	***Aedes aegypti* F**	***Aedes aegypti* M**	***Aedes albopictus* F**	***Aedes albopictus* M**	***Aedes caspius* F**	***Culiseta longiareolata* F**
Larnaca	CO_2	126	27	4	0	2	15	4	0	0	2	0
Nicosia	CO_2	305	10	4	0	2	0	0	53	16	0	1
Larnaca	CO_2 + BG Lure	96	16	5	1	3	51	7	0	0	3	0
Nicosia	CO_2 + BG Lure	318	16	1	0	1	0	0	80	33	0	0
Larnaca	BG Lure	16	36	0	4	0	26	7	0	0	0	0
Nicosia	BG Lure	18	10	0	0	1	0	0	71	52	0	1
Larnaca	None	9	23	0	0	0	19	4	0	0	0	0
Nicosia	None	18	7	0	0	0	0	0	62	54	0	2
